# Playing Roles in Work and Family: Effects of Work/Family Conflicts on Job and Life Satisfaction Among Junior High School Teachers

**DOI:** 10.3389/fpsyg.2021.772025

**Published:** 2021-12-20

**Authors:** Xing Li, Xinyue Lin, Fan Zhang, Yuan Tian

**Affiliations:** ^1^School of Business and Management, Shanghai International Studies University, Shanghai, China; ^2^Key Laboratory of Adolescent Cyberpsychology and Behavior (CCNU), Ministry of Education, School of Psychology, Central China Normal University, Wuhan, China; ^3^School of Psychology, Central China Normal University, Wuhan, China

**Keywords:** work to family conflict, family to work conflict, psychological capital, emotional exhaustion, job satisfaction, life satisfaction

## Abstract

Junior high school teachers play an essential role in education. How to relieve the worries of teachers, that is, the pressure they face in the fields of work and family, has increasingly become an urgent problem. Based on the COR theory, this study aims to investigate the effects of two types of work/family conflicts (i.e., work-family conflict and family-work conflict) on teachers’ job and life satisfaction. We adopted a handy sample method and collected a total of 560 junior high school teachers data. The results confirmed that both work-family and family-work conflicts are not significantly related to junior high school teachers’ job satisfaction and life satisfaction directly. However, both work-family and family-work conflicts have significantly negatively influence on junior high school teachers’ job satisfaction and life satisfaction via psychological capital and emotional exhaustion. Our findings provide new suggestions on how to alleviate the conflicts between work and family faced by teachers and further improve their satisfaction about work and family.

## Introduction

Education is one of the cornerstones of human development (Hofman, 2015; Madsen, 2020), and teachers, as a key pillar of the teaching process, have a very important impact on student achievement and school quality (Scheopner, 2010; Greaves and Sibieta, 2019; Motegi and Oikawa, 2019; Pozo-Rico and Sandoval, 2020). Their mental health have been paid much attention by researchers (Chirico, 2016; Zinsser et al., 2016; Shami et al., 2017). In the current manuscript, we pay particular attention to the group of junior high school teachers. Junior high school students are at their adolescence stage, when their body, mind, and values are developing rapidly (Lane, 2012; Chen et al., 2019; Nakano et al., 2019; Wan, 2021). The work attitude, behaviors and mental health of teachers at this crucial stage may have long-term impact on these sensitive students (Lane, 2012; Cuypers, 2017). However, studies have shown that junior high school teachers are facing with higher level of work/family conflict (Tanoi et al., 2012; Hu et al., 2016; Sorensen et al., 2017; Asriani, 2018), which may negatively affect their mental health (Hu et al., 2016; Ahmad and Islam, 2019; Bai et al., 2021) and further influence the quality of what they teach their students (Lane, 2012; Cuypers, 2017; Gu and Wang, 2021).

Work and family are two essential parts of an individual’s adult life (Sorensen et al., 2017; Asriani, 2018; French et al., 2018). For teachers, they should not only bear the heavy responsibility given to them by the country and society, but also bear the due obligations and responsibilities for their families (Makau, 2012; Sorensen et al., 2017). However, individual energy and time are always limited (Greenhaus and Beutell, 1985; Zhou et al., 2020; Delgado-Bello et al., 2021). Greenhaus and Beutell (1985) points out that work/family conflicts are the specific manifestation of role conflict, that is, the demands from the work and family fields are incompatible with each other. According to the conservation of resource theory (COR; Hobfoll, 1989), individuals’ psychological resources are limited. That is, once teachers put too much resources to the family domain, then resources investment in work may be affected, thus experiencing a decrease in job satisfaction. On the contrary, when more resources are invested in the work-related field in order to make progress in work, it inevitably leads to the reduction of the input of resources in the family field, thus decreasing family satisfaction (Shockley et al., 2017). Therefore, when there are conflicts between work and family, it is difficult for junior high school teachers to feel high job satisfaction or family satisfaction (Tanoi et al., 2012; Hu et al., 2016; Sorensen et al., 2017). Teachers’ satisfaction with their work and life will affect their enthusiasm for teaching (Gu and Wang, 2021). How to alleviate the conflict between the different roles of teachers from work and family and improve the perception of teachers’ satisfaction with work and family is thus increasingly urgent.

The bidirectionality of the relationship between work and family domains has been neglected in existing relevant studies. That is, scholars only examine the impact of work roles on family roles (i.e., WFC) (Grzywacz and Butler, 2005; Fiaz and Qureshi, 2021), or the impact of family roles on work roles (i.e., FWC) (Mcnall et al., 2009; Boz et al., 2016), or integrate them into one factor (Kelly and Tranby, 2011; Zhou et al., 2020; Fiaz and Qureshi, 2021). But in fact, WFC and FWC have different effects on job variables (e.g., job satisfaction) and non-job variables (e.g., marital satisfaction) (Netemeyer et al., 2004; Netemeyer and Pullig, 2005; Schenewark and Dixon, 2012; Ahmad and Islam, 2019). Therefore, it is necessary to explore the two types of work/family conflict separately and simultaneously.

With the prosperity of positive psychology in recent years, individual positive psychological energy has gained much attention in psychological field. Psychological capital is a kind of positive psychological resources which is highly valued (Luthans et al., 2007, 2010; Anglin et al., 2018). Existing studies have shown that work/family conflicts significantly negatively predict individual psychological capital (Jian and Sun, 2013; Lee and Gyum, 2013), which can significantly positively predict job satisfaction (Cheung et al., 2011; Karatepe and Karadas, 2014). More importantly, psychological capital is characterized as a state-like construct, which is confirmed the possibility of human intervention of psychological capital, such as the psychological capital intervention model (PCI) proposed by Luthans et al. (2007). According to the COR theory (Hobfoll, 1989), psychological capital, as an important psychological resource, must be an important resource for individuals to cope with work/family conflicts.

In addition, teachers’ emotional state is also worth taking into account when we explore the effects of work/family conflicts. Work/family conflicts are a source of stress for individuals to play different roles but fail to meet the requirements of roles at the same time (Bai et al., 2021; Delgado-Bello et al., 2021). Studies have shown that individuals with blurred role boundaries and high role conflict are more likely to experience emotional exhaustion (Liu et al., 2015). Moreover, emotional exhaustion can negatively affects life or job satisfaction (Boekhorst et al., 2017; Anastasiou and Belios, 2020). This study thus introduce psychological capital and emotional exhaustion as our potential mediators in the relationship between work/family conflicts and job satisfaction and life satisfaction, which can provide new perspectives for schools and relevant departments to alleviate work/family conflicts and improve teachers’ satisfaction with work and life.

**FIGURE 1:**
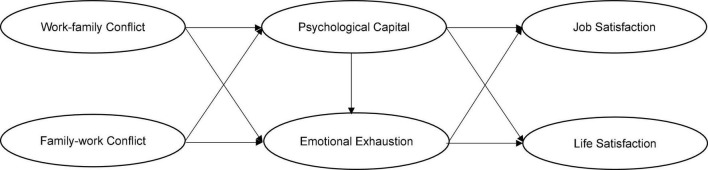
The theoretical model.

In sum, this study intends to investigate the effects of work-family conflict and family-work conflict on job satisfaction and life satisfaction among junior high school teachers, as well as the mediating effects of psychological capital and emotional exhaustion based on the COR theory. The theoretical model is presented in Figure 1.

## Theoretical Framework and Hypotheses Development

### Work-Family and Family-Work Conflicts

Family are always defined as a larger, non-work entity that includes responsibilities for spouse, children, and general family life (Netemeyer and Pullig, 2005; Delgado-Bello et al., 2021; Fiaz and Qureshi, 2021). Regardless of considering work and family as two relatively independent fields in early studies, researchers have increasingly pointed out their interdependence of each other (Edwards and Rothbard, 2000; Erdamar and Demirel, 2016; Zhou et al., 2020; Delgado-Bello et al., 2021). Spillover occurs when one domain (i.e., work or family) affects another domain (Grzywacz et al., 2002). Studies have shown that emotions, values, skills, behaviors (Edwards and Rothbard, 2000), attitudes, ideas, principles (Stevanovic and Rupert, 2009), stress and beliefs (Kinnunen et al., 2006), and other factors can be transferred from one domain to another (Hammer et al., 2005; Qureshi et al., 2019). Spillover can be divided into two forms: positive and negative (Grzywacz and Marks, 2000; Hammer et al., 2005; Carlson et al., 2006; Lee et al., 2021). Positive spillover refers to participation in an environment that benefits or promotes another area, creates satisfaction and enjoyment rather than stress (Rothbard, 2001; Hammer et al., 2005; Lee et al., 2021), and improves the quality of life in another area (Greenhaus and Powell, 2006). Conversely, negative spillover occurs when participation in one area makes it more difficult to participate in another (Greenhaus and Beutell, 1985; Leea et al., 2019; Lee et al., 2021). Specifically, meeting the needs of an individual in one area leads to greater stress in meeting the needs of the other area (Hammer et al., 1998). Negative spillover includes two specific forms: work-family conflict (WFC) and family-work conflict (FWC).

Kahn et al. (1964) defined work/family conflicts as the simultaneous demand of two or more roles conflict in some aspects. Work/family conflicts are manifestation of inter-role conflicts, in which the functional pressures from the work and family fields are incompatible in some aspects (Greenhaus and Beutell, 1985). That is, given the limited time and energy, individuals cannot meet the requirements of both work and family at the same time. It is thus difficult for individuals to adapt to the role of family (work) after fulfilling the role of work (family). Frone et al. (1992) pointed out that the conflict between work and family not only affects family life, but also work life, that is, the bidirectional nature of conflict. The interference from Work to Family is called *WFC (Work-Family Conflict)*, while the interference from Family to Work is called *FWC (Family-Work Conflict)*. For the sake of simplicity, the manuscript will collectively refer to work/family conflicts.

Based on the conservation of resource theory (COR, Hobfoll, 1989), previous studies believed that negative spillovers occur because individuals only have a certain amount of energy and resources, and participation in multiple fields will lead to energy consumption and resource reduction, which thus creating conflict and tension (Rothbard, 2001; van Steenbergen et al., 2014). From the perspective of resources, the current study is aimed to explore the mechanism of work/family conflicts affecting individual work and life based on the COR theory.

### Work/Family Conflicts, Job Satisfaction, and Life Satisfaction

According to the COR theory, individuals always want to maintain, protect and construct the resources that they feel are valuable. If these resources are threatened by loss, they will feel pressure (Hobfoll, 1989). For individuals, when they encounter conflict between work and family, they will inevitably consume their own resources to cope with the conflict. Individuals are aware of the continuous loss of their own resources, without the replenishment of resources (Halbesleben et al., 2014). The continuous consumption will make individuals feel pressure, and they will be unable to better undertake the responsibilities in work or family. This, in turn, has brought negative effects on several outcomes, mainly including work-related outcomes (e.g., job satisfaction and job performance) and non-work-related outcomes (e.g., life satisfaction and family satisfaction) (Bruck et al., 2002; Brough et al., 2005; McNall et al., 2010; French et al., 2018; Gu and Wang, 2021). Among these outcomes, in particular, we focus on teachers’ well-being outcomes, that is, job satisfaction and life satisfaction, both of which are crucial factors for junior high school teachers.

Job satisfaction refers to the subjective satisfaction perceived by employees in terms of work content, work experience, and work environment (Malinen and Savolainen, 2016; Gil-Flores, 2017; Reeves et al., 2017). Extant studies pointed out there are mainly two types of factors influencing job satisfaction: individual characteristics and organizational environment (Wanjagua, 2012; Larkin, 2016). First, teachers’ job satisfaction is different in terms of age, marital status and other aspects. For example, the job satisfaction of unmarried teachers is generally higher than that of married teachers (Nayak and Nayak, 2014). In terms of organizational environment, teachers’ job satisfaction is affected by negative factors, such as burnout (Smetackova et al., 2019), stress (Mlyuka, 2015; Luturlean et al., 2019; Delgado-Bello et al., 2021), and conflict (Makau, 2012; Palomino and Frezatti, 2016). As a combination of work stress and family stress, relevant studies on teachers have found that both dimensions of work/family conflicts have a significant negative correlation with job satisfaction (Makau, 2012; Gao et al., 2013; Erdamar and Demirel, 2016; Delgado-Bello et al., 2021). Conflicts lead to an increase in individuals’ perceived negative emotions while playing corresponding roles in different domains, which further reduce their satisfaction in these domains (Frone et al., 1992; Aryee et al., 2008; Makau, 2012; Palomino and Frezatti, 2016; Delgado-Bello et al., 2021).

Life satisfaction refers to an individual’s overall evaluation of his/her life quality (Lambert, 2010; Corrigan et al., 2013; Erdamar and Demirel, 2016), which influenced by life experience, interpersonal relationship, family environment (Erdamar and Demirel, 2016), demographic factor (e.g., gender) (Kates, 2007), and the relationship between work and family (Erdamar and Demirel, 2016). Studies exploring conflict and life satisfaction show that both types of work-family conflict have a significant negative correlation with life satisfaction (Mesmer-Magnus and Viswesvaran, 2005; Naz et al., 2011; Bai et al., 2021). Therefore, we propose that:

*Hypothesis 1a: Work-family conflict is negatively related to job satisfaction and life satisfaction.*

*Hypothesis 1b: Family-work conflict is negatively related to job satisfaction and life satisfaction.*

### The Mediating Role of Psychological Capital

With an increasing recognition of the value of positive organizational behavior, organizations sought to improve employees’ physical and psychological health by strengthening the psychological resources of employees (Wang et al., 2012). Appearing first in economics fields, psychological capital has gained its popularity in positive psychology field (Anglin et al., 2018). Csikszentmihalyi and Seligman (2000) believe that psychological factors that may promote individuals to produce positive behaviors should be included in the category of psychological capital. Luthans and Youssef (2004) then put forward the concept of psychological capital from the perspective of positive organizational behavior. They pointed out that psychological capital is a kind of stable and changeable positive psychological energy displayed by individuals in the process of their growth and development (Luthans et al., 2007). Characterizing as a state-like construct, psychological capital contains four dimensions, namely self-efficacy, optimism, hope, and resilience (Luthans et al., 2007).

We propose that individual psychological capital may be affected by work/family conflicts. Work/family conflicts are caused by incompatible pressures from work and family fields, that is to say, individual time and energy cannot meet the requirements of two fields at the same time (Greenhaus and Beutell, 1985; Pu et al., 2017). Therefore, the contradictions and conflicts between work and family will inevitably cause the consumption of individual psychological resources. According to the COR theory (Hobfoll, 1989), psychological capital, as an important psychological resource, must be an important resource for individuals to cope with work/family conflicts. Meanwhile, since psychological capital is a state-like construct (Luthans et al., 2007), individuals will experience the continuous conflict and contradiction between work and family, which will lead to the decline of their psychological capital level. On the other hand, psychological capital is closely related to job satisfaction and life satisfaction. Extant studies have shown that psychological capital, as a kind of positive psychological energy, can improve job and life satisfaction by improving job engagement (Cheung et al., 2011; Karatepe and Karadas, 2014). Taking together, we hypothesize that psychological capital mediates the relationships between work/family conflicts and job/life satisfaction.

*H2a: Psychological capital mediates the relationship between work-family conflict and job satisfaction and life satisfaction.*

*H2b: Psychological capital mediates the relationship between family-work conflict and job satisfaction and life satisfaction.*

### The Mediating Role of Emotional Exhaustion

American psychologist Freudenberger (1975), in his research related to nursing staff, put forward the concept of job burnout to describe a series of psychological and physiological syndromes caused by continuous work stress and interpersonal stress among service industry practitioners. Maslach et al. (2001) believe that job burnout is a state of physical, mental, emotional, emotional and behavioral exhaustion caused by too long working hours, too much work, too much work intensity, and disregard for one’s own needs. Job burnout can be divided into three dimensions: emotional exhaustion, de-individuation, and low sense of achievement (Maslach et al., 2001). Among them, emotional exhaustion refers to a state of fatigue caused by the exhaustion of emotional resources (Jackson et al., 1986; Maslach and Jackson, 1986; Kahill, 1988). We especially focus on emotional exhaustion. With the deepening of research on job burnout, different researchers have pointed out that emotional exhaustion is the most prominent manifestation of job burnout, presenting fatigue caused by excessive work requirements (Moon and Hur, 2011; Mao et al., 2020). It is a feeling of excessive emotional consumption of individuals, leading to a variety of negative symptoms and feelings in both physical and psychological aspects (Liu et al., 2015; Boekhorst et al., 2017; Anastasiou and Belios, 2020).

Existing studies have shown that emotional exhaustion has a significant negative predictive effect on both physical and mental health of individuals (Liu et al., 2015; Boekhorst et al., 2017; Anastasiou and Belios, 2020; Mao et al., 2020). In addition, emotional exhaustion also affects employees’ attitudes toward work or life (Boekhorst et al., 2017; Anastasiou and Belios, 2020). Under the state of continuous emotional exhaustion, individuals will gradually lose their enthusiasm for work, complain about the organizations they work for, and their job satisfaction will decrease, and it will be difficult for them to devote themselves to their work (Cropanzano et al., 2003). Emotional exhaustion also can affect individuals’ life. Webster et al. (2010) pointed out that the negative effects of emotional exhaustion on individuals can spread to areas outside of work, and the higher the level of emotional exhaustion, the lower the life satisfaction of individuals.

As for the junior high school teachers, the social requirements for teachers’ work are increasing day by day, which leads to the increase in resources required by teachers, such as energy, time, and emotion (Ye and Ye, 2015). Due to the particularity of teachers’ work, that is, the high requirements of teaching work cannot be met during working hours, teachers have to divert resources that should be paid for family life, which leads to the situation that work interferes with family life. Specially, due to the different division of labor assigned by the society, for female teachers, they may have more family responsibilities, which will consume a lot of their resources, and sometimes even need to take up their working time to deal with family affairs, resulting in family interference to work. Empirical studies also show that work/family conflicts are significant negative predictor of emotional exhaustion (Hall et al., 2011; Leineweber et al., 2012). Taking together, we hypothesize that emotional exhaustion mediates the relationships between work/family conflicts and job/life satisfaction.

*H3a:* Emotional exhaustion *mediates the relationship between work-family conflict and job satisfaction and life satisfaction.*

*H3b:* Emotional exhaustion *mediates the relationship between family-work conflict and job satisfaction and life satisfaction.*

Meanwhile, extant research also shows that the decrease of psychological capital level will also increase individual emotional exhaustion level (Yin et al., 2018; Khalid et al., 2020). According to the COR theory, tasks and various difficulties faced by individuals at work are important sources of work pressure (Hobfoll, 1989; Ford, 2007; Halbesleben et al., 2014), which requires the mobilization of resources from all aspects of individuals to cope with, and continuous physical and psychological efforts. If individuals cope with difficulties and resources are limited, they will experience high levels of stress, and the continuous consumption of physical and mental resources will lead to exhaustion (Boekhorst et al., 2017; Anastasiou and Belios, 2020). Therefore, individual psychological capital levels may affect the degree of emotional exhaustion, and then having a negative impact on individual job satisfaction and life satisfaction. Therefore, we further propose that:

*H4a: Psychological capital and emotional exhaustion mediate serially the relationships between work-family conflict and job satisfaction and life satisfaction.*

*H4a: Psychological capital and emotional exhaustion mediate serially the relationships between family-work conflict and job satisfaction and life satisfaction.*

## Materials and Methods

### Participants and Procedures

We conducted an online survey and collected data on full-time teachers from different junior high schools in central China. We adopted a handy sample method by using existing social networks to collecting data. A total of 560 questionnaires were returned. After excluding the uncompleted questionnaires, 515 valid questionnaires were retained. The effective rate of the questionnaire was 91.9%. The demographic information about the sample is shown in Table 1. The ratio of men to women is relatively even (45% male and 55% female), as well as the age range (24.7% for 30 years old or below, 30.9% for 31–40 years old, and 33.2% for 41–50 years old). Most of the subjects are married (80.2% are married), which fits our survey situation. In terms of education level, participants mainly have a bachelor’s degree (85.1%). 54.6% of teachers have at least 16 years of teaching experience.

**TABLE 1:** Demographic results.

		*N*	Percentage (%)
Gender	Male	232	45.0
	Female	283	55.0
Age	≤30 years old	127	24.7
	31–40 years old	159	30.9
	41–50 years old	171	33.2
	≥51 years old	58	11.3
Marital status	Married	413	80.2
	Unmarried	93	18.1
	Divorced	9	1.7
Education background	Junior college and below	34	6.6
	Undergraduate	438	85.0
	Master and above	43	8.3
Class adviser	Yes	217	42.1
	No	298	57.9
Working time	≤5 years	120	23.3
	6–10 years	58	11.3
	11–15 years	56	10.9
	≥16 years	281	54.6
Work location	City	147	28.5
	Town	176	34.2
	Village	192	37.3
School rank	Key	134	26.0
	General	381	74.0
Teaching grade	Grade 7	185	35.9
	Grade 8	157	30.5
	Grade 9	173	33.6
			

### Measures

The questionnaire was designed based on previously validated measurement scales. The English original scales were translated to Chinese using Brislin’s (1986) translation and back-translation procedures. We adopted a five-point Likert scale, with “1” to “5” meaning “strongly disagree” to “strongly agree.”

#### Work/Family Conflicts

The scale developed by Wu et al. (2009) was adopted. The scale contains two subscales to measure two types of work/family conflicts. Each subscale contains 11 items to measure behavioral conflict, resource conflict and emotional conflict dimensions. The higher the score, the more serious the conflicts. Sample items read: “Things at work put me in a bad mood and affected the family atmosphere” for work-family conflict; “The conflict with my family members put me in a bad mood at work” for family-work conflict. The Cronbach’s α for work-family conflict and family-work conflict are 0.92 and 0.91, respectively.

#### Job Satisfaction

The six-item scale developed by Tsui et al. (1992) was adopted. A higher total score indicates a higher degree of job satisfaction. A sample item is “I am satisfied with my work environment considering every aspect.” Cronbach’s α = 0.85.

#### Life Satisfaction

The five-item scale developed by Pavot and Diener (2009) was adopted. The higher the total score, the higher the teacher’s life satisfaction. The scale has been validated under multiple national contexts. A sample item is “I am satisfied with my life.” Cronbach’s α = 0.88.

#### Psychological Capital

The scale developed by Luthans et al. (2007) was adopted. The scale contains 24 items to measure four dimensions, with 6 items for each dimension. Among them, three questions were designed for reverse scoring. The higher the overall score, the higher the degree of psychological capital. Sample items include “I believe I can analyze long-term problems and find solutions” and “I can think of many ways to achieve my current goal.” Cronbach’s α for the whole scale is 0.92.

#### Emotional Exhaustion

The seven-item emotional exhaustion subscale in the Teacher Burnout Questionnaire developed by Xu et al. (2004) was adopted. The higher the total points, the higher the degree of emotional exhaustion. A sample item is “I feel physically and mentally exhausted.” Cronbach’s α = 0.87.

### Analytical Strategy

We used SPSS 22.0 software to test common method variance, descriptive statistical analysis of variables and correlation analysis on the collected data, and used Mplus 7.0 to conduct structural equation modeling to test the mediation mechanism of psychological capital and emotional exhaustion.

Since the data used in the study were all self-reported, there may be common method bias, which is a systematic error reducing the validity of research. In order to avoid common method deviation as far as possible, we first control the test procedure such as the use of reverse scoring. Secondly, we conducted Harman single factor test, and the results showed that among the factors without rotation, there were 13 factors whose characteristic root was greater than 1, among which the variance explained by the first factor was 18.6% (less than 40%). Therefore, the problem of common method bias of data in this study is not serious.

## Results

### Confirmatory Factor Analysis

We pay attention to understanding the relationships between variables. We thus used the parceling method in fitting our model to give us a better fit index (Kline, 2005), considering the fact that certain focal variables have relatively more items. We first packaged each variable, that is, work-family conflict with three indicators; family-work conflict with three indicators; psychological capital with four indicators; emotional exhaustion with three indicators; both job satisfaction and life satisfaction with three indicators. We then conducted confirmatory factor analysis among a total of six latent variables. The results show that the measurement model of this study fits well: χ^2^ (72, *N* = 515) = 531.78, CFI = 0.94, TLI = 0.92, SRMR = 0.05, RMSEA = 0.07, 90% confidence interval of RMSEA is [0.07, 0.08]. As shown in Table 2, the standardized load of each indicator on its corresponding factor was significant (*p* < 0.001).

**TABLE 2:** Factor loadings of latent variables in measurement models.

Latent variables	Unstandardized factor loading	*SE*	*t*	Standardized factor loading
**Work-family conflict**				
Resource conflict	1.00	0.00	999.00	0.85***
Emotion conflict	0.99	0.05	18.71	0.74***
Behavior conflict	1.24	0.05	23.46	0.87***
**Family-work conflict**				
Resource conflict	1.00	0.00	999.00	0.76***
Emotion conflict	1.18	0.07	17.38	0.77***
Behavior conflict	1.37	0.07	20.01	0.91***
**Psychological capital**				
Self-efficacy	1.00	0.00	999.00	0.78***
Hope	1.21	0.06	20.53	0.87***
Resilience	0.89	0.05	17.91	0.76***
Optimism	0.89	0.05	17.86	0.79***
**Emotional exhaustion**				
Item 1	1.00	0.00	999.00	0.80***
Item 2	1.30	0.07	18.40	0.82***
Item 3	1.13	0.06	18.00	0.78***
**Job satisfaction**				
Item 1	1.00	0.00	999.00	0.84***
Item 2	1.17	0.06	21.20	0.86***
Item 3	1.14	0.06	17.83	0.73***
**Life satisfaction**				
Item 1	1.00	0.00	999.00	0.85***
Item 2	0.88	0.04	223.63	0.87***
Item 3	0.94	0.04	22.53	0.85***

***p < 0.001.

### Descriptive Statistics and Correlations

Table 3 presents the mean, standard deviation and correlations among the focal variables. The results showed that the junior high school teachers’ work-family conflict (*M* = 3.09, *t* = −2.36, *p* < 0.05) was above the median value (hypothesizing the median value of 3), while the level of family-work conflict (*M* = 2.51, *t* = −14.25, *p* < 0.01) was lower than the median value. Likewise, the level of psychological capital of junior high school teachers (*M* = 3.64, *t* = 27.87, *p* < 0.01) was above the median value, while the emotional exhaustion level (*M* = 2.71, *t* = −7.86, *p* < 0.01) of junior high school teachers was below the median value. The overall level of job satisfaction of primary and secondary school teachers (*M* = 3.10, *t* = 3.27, *p* < 0.01) was above the median value. The life satisfaction of junior high school teachers (*M* = 2.90, *t* = −2.75 *p* < 0.01) was lower than the median value. In addition, we got the expected correlation results among these six variables.

**TABLE 3:** Means, standardized deviation, and correlation results.

Variables	*M*	*SD*	1	2	3	4	5	6	7
1 Work/family conflicts	2.80	0.75	1						
2 Work-family conflict	3.09	0.85	0.93**	1					
3 Family-work conflict	2.51	0.78	0.92**	0.70**	1				
4 Psychological capital	3.64	0.52	−0.34**	−0.30**	−0.34**	1			
5 Emotional exhaustion	2.71	0.85	0.57**	0.56**	0.50**	−0.44**	1		
6 Job satisfaction	3.10	0.71	−0.36**	−0.39**	−0.27**	0.40**	−0.51**	1	
7 Life satisfaction	2.90	0.86	−0.30**	−0.35**	−0.20**	0.42**	−0.41**	0.60**	1

***p < 0.001.

### Hypotheses Testing

We then used Mplus7.0 to conduct structural equation modeling to test the mediation mechanism of psychological capital and emotional exhaustion between the relationship between two types of conflicts and two types of satisfactions.

**FIGURE 2:**
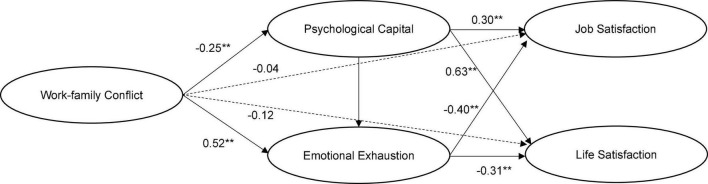
The effects of work-family conflict. **p < 0.01. Latent variables only are presented to make the model look concise. The same as below.

The results were shown in Figure 2. The data fit the model well (χ^2^ = 381.5, χ^2^/df = 3.50, RMSEA = 0.07, SRMR = 0.05, CFI = 0.95, TLI = 0.93). The direct effect of work-family conflict on teachers’ job satisfaction is not significant (γ = −0.04, *t* = −0.59, *p* > 0.05), nor is the direct effect on teachers’ life satisfaction (γ = −0.12, *t* = −1.17, *p* > 0.05). However, work-family conflict has a significant negative impact on psychological capital (γ = −0.25, *t* = −6.15, *p* < 0.01), and psychological capital has a significant positive impact on job satisfaction (γ = 0.30, *t* = −3.21, *p* < 0.01). Psychological capital mediates the relationship between work-family conflict and teachers’ job satisfaction (indirect effect = −0.08, *p* < 0.05). Psychological capital also has a significant positive impact on life satisfaction (γ = 0.63, *t* = 4.86, *p* < 0.01), which indicates that psychological capital also mediates the relationship between work-family conflict and teachers’ life satisfaction (indirect effect = −0.16, p < 0.05). Hypothesis 2a is verified. Similarly, work-family conflict has a significant positive effect on emotional exhaustion (γ = 0.52, *t* = 8.34, *p* < 0.01), and emotional exhaustion has a significant negative effect on job satisfaction (γ = −0.40, *t* = −4.89, *p* < 0.01). Emotional exhaustion mediates the relationship between work-family conflict and teachers’ job satisfaction (indirect effect = −0.21, *p* < 0.05). Emotional exhaustion also has a significant negative impact on life satisfaction (γ = 0.63, *t* = 4.86, *p* < 0.01), which indicates that emotional exhaustion also mediates the relationship between work-family conflict and teachers’ life satisfaction (indirect effect = −0.16, *p* < 0.05). Hypothesis 3a is verified.

In addition, work-family conflict also had a significant indirect effect on job satisfaction through the chain mediation effects of psychological capital and emotional exhaustion (indirect effect = −0.05, *p* < 0.05), as well as on having a significant indirect effect on life satisfaction through the chain mediation effects of psychological capital and emotional exhaustion (indirect effect = −0.03, *p* < 0.05). Hypothesis 4a is verified. Further, the bias corrected percentile Bootstrap analysis method was used to test the significance of the above mediation effects. The results in Table 4 showed that the 95% confidence intervals of the above mediation paths do not include 0, indicating that they are all significant mediating effects.

**TABLE 4:** Bootstrapped results for the mediators in the effects of work-family conflict.

Path	Indirect effect	95% confidence interval	
		Lower 2.5%	Upper 2.5%
Work-family conflict → Psychological capital → Job satisfaction	−0.08	−0.148	−0.030
Work-family conflict → Psychological capital → Life satisfaction	−0.16	−0.185	−0.069
Work-family conflict → Emotional exhaustion → Job satisfaction	−0.21	−0.363	−0.135
Work-family conflict → Emotional exhaustion → Life satisfaction	−0.16	−0.236	−0.021
Work-family conflict → Psychological capital → Emotional exhaustion → Job satisfaction	−0.05	−0.083	−0.026
Work-family conflict → Psychological capital → Emotional exhaustion → Life satisfaction	−0.03	−0.051	−0.004

**FIGURE 3:**
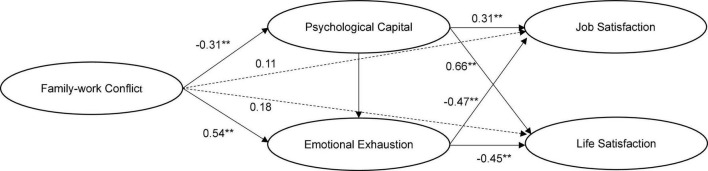
The effects of family-work conflict. **p < 0.01.

Likewise, the theoretical model considering the effect of family-work conflict fit well (χ^2^ = 328.2, χ^2^/df = 3.01, RMSEA = 0.06, SRMR = 0.05, CFI = 0.96, TLI = 0.94). As shown in Figure 3, the direct effect of family-work conflict on teachers’ job satisfaction is not significant (γ = 0.11, *t* = 1.71, *p* > 0.05), nor is the direct effect on teachers’ life satisfaction (γ = 0.18, *t* = 1.85, *p* > 0.05). However, family-work conflict has a significant negative impact on psychological capital (γ = −0.31, *t* = −5.68, *p* < 0.01), and psychological capital has a significant positive impact on job satisfaction (γ = 0.31, *t* = −3.34, *p* < 0.01). Psychological capital mediates the relationship between family-work conflict and teachers’ job satisfaction (indirect effect = −0.10, *p* < 0.05). Psychological capital also has a significant positive impact on life satisfaction (γ = 0.66, *t* = 5.04, *p* < 0.01), which indicates that psychological capital also mediates the relationship between family-work and teachers’ life satisfaction (indirect effect = −0.20, *p* < 0.05). Hypothesis 2b is verified. Similarly, family-work conflict has a significant positive effect on emotional exhaustion (γ = 0.54, *t* = 7.08, *p* < 0.01), and emotional exhaustion has a significant negative effect on job satisfaction (γ = −0.47, *t* = −6.57, *p* < 0.01). Emotional exhaustion mediates the relationship between family-work conflict and teachers’ job satisfaction (indirect effect = −0.31, *p* < 0.05). Emotional exhaustion also has a significant negative impact on life satisfaction (γ = −0.45, *t* = −4.17, *p* < 0.01), which indicates that emotional exhaustion also mediates the relationship between family-work conflict and teachers’ life satisfaction (indirect effect = −0.24, *p* < 0.05). Hypothesis 3b is verified.

In addition, family-work conflict also had a significant indirect effect on job satisfaction through the chain mediation effects of psychological capital and emotional exhaustion (indirect effect = −0.07, *p* < 0.05), as well as on having a significant indirect effect on life satisfaction through the chain mediation effects of psychological capital and emotional exhaustion (indirect effect = −0.07, *p* < 0.05). Hypothesis 4b is verified. Further, the bias corrected percentile Bootstrap analysis method was used to test the significance of the above mediation effects. The results in Table 5 showed that the 95% confidence intervals of the above mediation paths do not include 0, indicating that they are all significant mediating effects.

**TABLE 5:** Bootstrapped results for the mediators in the effects of family-work conflict.

Path	Indirect effect	95% confidence interval	
		Lower 2.5%	Upper 2.5%
Family-work conflict → Psychological capital → Job satisfaction	−0.10	−0.157	−0.032
Family-work conflict → Psychological capital → Life satisfaction	−0.20	−0.197	−0.072
Family-work conflict → Emotional exhaustion → Job satisfaction	−0.31	−0.343	−0.153
Family-work conflict → Emotional exhaustion → Life satisfaction	−0.24	−0.246	−0.073
Family-work conflict → Psychological capital → Emotional exhaustion → Job satisfaction	−0.07	−0.105	−0.037
Family-work conflict → Psychological capital → Emotional exhaustion → Life satisfaction	−0.07	−0.073	−0.018

## Discussion

Based on the COR theory, this study aims to investigate the effects of two types of work/family conflicts (i.e., work-family conflict and family-work conflict) on teachers’ job and life satisfaction. The results from 560 of junior high school teachers data collected by online survey confirmed that both work-family and family-work conflicts are not significantly related to junior high school teachers’ job satisfaction and life satisfaction directly. However, both work-family and family-work conflicts are significantly negatively related to junior high school teachers’ job satisfaction and life satisfaction via psychological capital and emotional exhaustion. This study has certain theoretical significance. Most of the previous studies have focused on the negative effects of work/family conflicts on single domains of work or family, and few have focused on the mechanism of work/family conflicts on both domains. This study explores and confirmed the relationship between work/family conflicts and job satisfaction and life satisfaction. Secondly, this study explores the internal mechanism of two different dimensions of work/family conflicts affecting work and life satisfaction, and investigates the chain mediating role of psychological capital and emotional exhaustion in the above relationship. It helps us understand more about how work/family conflict affects work and life satisfaction.

### The Mediation Role of Psychological Capital

First of all, psychological capital plays a mediating role in the relationship between work-family conflict and job and life satisfaction. This result indicates that because teachers in primary and secondary schools have long time and heavy workload for a long time, their job satisfaction will inevitably decrease. Due to the particularity of the teaching profession, it is inevitable to take work home to deal with (Cinamon et al., 2007). Working long hours and dealing with related affairs at home lead to the decline of life satisfaction. Psychological capital refers to the positive psychological energy of an individual (Luthans and Youssef, 2004). In the face of the negative situation of work-family conflict, an individual will use psychological capital to deal with this negative situation, which will also lead to the consumption of psychological capital (Luthans et al., 2007), and then affect the job and life satisfaction of primary and secondary school teachers.

Psychological capital also play a mediating role in the relationship between family-work conflict and satisfaction with work and life. Existing studies have shown that family-work conflict is closely related to individual occupational mental health, such as job satisfaction, job distress, and absenteeism rate (Bagger et al., 2008; Karatepe and Karadas, 2014; Biricik, 2020). The imbalance between work and family is also one of the important stressors to reduce individual career happiness (Bellavia and Frone, 2005). Home-based teachers have to take care of children, deal with the relationships between husbands or wives, as well as the relationships with the elders (e.g., parents-in-law) in the extended family (Baba, 2018; Noor and Isa, 2020; Park and Park, 2019). All these aspects will consume the individual’s psychological capital, thus leading to the reduction of work and life satisfaction.

### The Mediation Role of Emotional Exhaustion

Secondly, the results of this study show that emotional exhaustion plays a mediating role in the impact of the two dimensions of work/family conflicts on teachers’ job and life satisfaction. If teachers are in a state of long-term imbalance between work and family, that is, paying too much time and energy in work and failing to fulfill their family responsibilities, they will attribute the reason to excessive pay in work (Bruck et al., 2002), resulting in rejection and boredom of work. The stress state will continue to increase, resulting in more anxiety, and the enthusiasm and motivation for work will continue to decline. Emotional exhaustion and job burnout will continue to occur, which will lead to the continuous impact of job satisfaction (Hakanen et al., 2006). Teachers experience a high level of emotional exhaustion in daily work, and this state will spill over into the family, teachers do not have extra resources to complete their family responsibilities, there will be conflicts and contradictions between husband and wife over time, which will lead to a decline in life satisfaction.

At the same time, emotional exhaustion also played a mediating role in the effects of family-work conflict on teachers’ job satisfaction and life satisfaction. As the breadwinner of the family, the individual needs to meet too many demands in the complicated family affairs, and cannot be said to be suffering. These situations lead to the emotional exhaustion of the individual, which leads to the reduction of work and life satisfaction.

### The Chain Mediation Role of Psychological Capital and Emotional Exhaustion

Finally, this study also found that psychological capital and emotional exhaustion play a chain mediating role in the relationship between the two types of work/family conflicts and junior high school teachers’ job satisfaction and life satisfaction, which was consistent with the view of the COR theory. First of all, due to the conflict between work and family, an individual needs to mobilize a large amount of resources to cope with the demands in each field. However, since the resources an individual has are limited, no matter which field an individual consumes a large amount of resources, it will affect the level of individual psychological capital. Second, if teachers constantly feel resource consumption (due to higher work requirements or higher family requirements), it is likely to lead to more resource depletion and affect the replenishment of resources. Over time, the resources available for coping with stress and conflict decrease, and leading to emotional exhaustion. The occurrence of emotional exhaustion will further affect the degree of teachers’ satisfaction with their work and life.

### Practical Implications

At the practical level, this study explores the different impacts of different dimensions of work/family conflicts on work and family, and suggests that we need to be vigilant about the conflict between teachers’ work and family in real life. In real life, teachers’ work and family conflict will always exist. However, when some people suffer from work/family conflicts, they may not always have a serious impact on their work or life satisfaction.

The research findings show that we may alleviate the negative effects of work/family conflicts by increasing individual psychological resources that can make individuals full of confidence and believe that they can deal with the current problems (Wang et al., 2012). From the positive psychology perspective, psychological capital is such one crucial psychological resource that need be emphasized by junior high school teachers and schools. Even though work/family conflicts may weaken psychological capital and thus undermine satisfaction, this influencing pathway can be weakened as long as individuals get relatively higher psychological capital. Psychological capital is a “state-like” construct and is open to be developed and managed (Luthans and Youssef, 2004). Previous studies have confirmed that the effectiveness of interventions designed to enhance components of psychological capital including self-efficacy, hope, resilience, and optimism (Lupşa et al., 2020). Therefore, strategies of enhancing junior high school teachers’ psychological capital should be developed.

Not only teachers should consciously exercise and improve psychological capital so as to make their own internal stronger in the face of the conflict between work and family, but also schools are supposed to give teachers more support. For example, where appropriate, by introducing flexible working hours so that they can spend time with their families. Flexible working hours are usually arrangements between an employer and an employee in which they agree to schedule the work flexibly, gaining benefits to both parties (Galea et al., 2014). Although teachers have lessons which must start and end at certain times but for the rest lessons, they can decide when, where and how they work. Flexible working hours are used because the lessons might not cover even the half of the work they do (Lahti, 2017). All preparations, reviewing exams and meetings with the students’ parents can and must be done using their own scheduling. Therefore, teachers can make time for life but perform the duties when it is most suitable for them (Lahti, 2017). Because of flexibility, it is possible to combine different roles in life and attain better work-life balance.

In addition, creating a good humanistic atmosphere by taking appropriate ways is also important, like holding group building activities to relieve teachers’ emotional tension and exhaustion, setting psychological counseling room where teachers who are under high pressure can seek counseling from psychological counselors in time to help solve their own problems.

### Limitations and Future Research

There are several limitations in this study. First of all, the data used in this study are cross-sectional, which to some extent cannot well explain the causal relationship between variables, and may lead to the distortion of the proportion of intermediate results (Maxwell and Cole, 2007). Future research should consider adopting longitudinal research methods to better reveal the relationship between variables. Secondly, the data used in this study are all from the self-evaluation of the subjects. Although reverse scoring and other methods as well as related statistical methods are used to control and test the possible deviation of the common method, the data collected from various aspects will undoubtedly make the research results more reliable. Ratings from other parties can give us a deeper understanding of variables and more objective data (Netemeyer et al., 2004).

## Data Availability Statement

The raw data supporting the conclusions of this article will be made available by the authors, without undue reservation.

## Ethics Statement

The studies involving human participants were reviewed and approved by the Ethic Institutional Review Board of Central China Normal University. The patients/participants provided their written informed consent to participate in this study.

## Author Contributions

XL and FZ contributed to conception and design of the study. XLin organized the database and performed the statistical analysis. XL wrote the first draft of the manuscript. YT reviewed the manuscript and supervised the whole project. All authors contributed to manuscript revision, read, and approved the submitted version.

## Conflict of Interest

The authors declare that the research was conducted in the absence of any commercial or financial relationships that could be construed as a potential conflict of interest.

## Publisher’s Note

All claims expressed in this article are solely those of the authors and do not necessarily represent those of their affiliated organizations, or those of the publisher, the editors and the reviewers. Any product that may be evaluated in this article, or claim that may be made by its manufacturer, is not guaranteed or endorsed by the publisher.
